# Hospital outbreak of extended-spectrum beta-lactamase-producing *Escherichia coli* potentially caused by toilet and bath chair use

**DOI:** 10.1016/j.infpip.2022.100239

**Published:** 2022-08-11

**Authors:** Naoto Okada, Mari Takahashi, Yumiko Yano, Masami Sato, Akane Abe, Keisuke Ishizawa, Momoyo Azuma

**Affiliations:** a Department of Pharmacy, Tokushima University Hospital, Tokushima, Japan; b Department of Infection Control and Prevention, Tokushima University Hospital, Tokushima, Japan; c Department of Clinical Pharmacology and Therapeutics, Tokushima University Graduate School of Biomedical Sciences, Tokushima, Japan; d Clinical Research Center for Developmental Therapeutics, Tokushima University Hospital, 2-50-1 Kuramoto, Tokushima, 770–8503 Japan

Dear Editor,

The worldwide spread of extended spectrum β-lactamase (ESBL)-producing bacteria, identified several decades ago, has become a serious concern in healthcare [1,2]. An important route of transmission of ESBL-producing bacteria in healthcare is via the healthcare environment. In particular, toilets have been reported to be a noteworthy infection source [3,4]. This study reports an outbreak in a 692-bed university hospital, in which the toilet and shower room bath chair were the potential sources of the transmission of ESBL-producing *Escherichia coli*. This outbreak may have been associated with inadequate cleaning protocols of the janitors.

In August 2021, ESBL-producing *E. coli* was detected in the urine and stool of two patients (Patients 1 and 2) in the same ward on 2 consecutive days. The infection prevention and control (IPC) team instructed the ward staff to adhere to appropriate hand hygiene [5] and to follow standard precautions as per the hospital IPC manual. However, 3 days later, ESBL-producing *E. coli* was detected in the stool of a third patient (Patient 3). We conducted a clonality investigation using the polymerase chain reaction-based open-reading frame typing (POT) method to confirm homology [6]. One *E. coli* strain was consistently detected in patients 1, 2, and 3 (POT number: 20-1-4), and this strain belonged to the CTX-M1 group. Subsequently, we performed environmental sampling of this ward, active surveillance to investigate the spread of ESBL-producing *E. coli* in hospitalised patients in this ward and introduced disinfection of environments with high frequency of contact using alcohol cloths. Active surveillance revealed ESBL-producing *E. coli* with the same POT typing in the stool of two patients (Patients 4 and 5). Patient 4 was subsequently diagnosed with bacteraemia caused by the outbreak strain. Eight other patients were found to carry ESBL-producing *E. coli*. However, their POT types did not match the outbreak strain. Hence, this was considered an outbreak in which the same ESBL-producing *E. coli* spread to a total of five patients.

A review of the patients' room history is shown in Figure 1A. All five patients' hospitalisations overlapped for 7 days. Patients 2, 3, and 5 were temporarily admitted in the same room. In the environmental samples, ESBL-producing *E. coli* with the same POT type as the outbreak strain was detected in the toilets used by patients 2, 3, and 5 and in the shower room bath chair used by all the patients. Hence, the toilet and the shower room were the possible sources for this outbreak. Although the janitor was responsible for cleaning toilets and shower room, the janitor continued to reuse disposable cleaning supplies and did not change these supplies between the different rooms and patient areas. The cleaning and drying of the reused mop heads was inadequate. Furthermore, janitors did not adhere to the hand hygiene guidelines when moving between different patient rooms and did not change gloves until cleaning was completed. No outbreak strains were detected in the cleaning supplies used by the janitor, but *Pseudomonas* spp., *Enterobacter* spp., and *Acinetobacter* spp. were detected. The IPC team concluded that the inadequate cleaning methods used by the janitor in this ward was the most likely the vector of the outbreak strain. The IPC team developed a manual specifying the correct hand hygiene practices for the janitors which prohibited the reuse of disposable cleaning equipment, and the IPC team provided education for the janitors about contact precautions. Subsequently, there was no further detection of the outbreak strain. A total of 53 patients hospitalised in this ward underwent follow-up screening during the next 3 months, and the outbreak strains were not detected in any of them. The numbers of samples positive for ESBL-producing *E. coli* in the ward before and after the outbreak are shown in Figure 1B.

**Figure 1:**
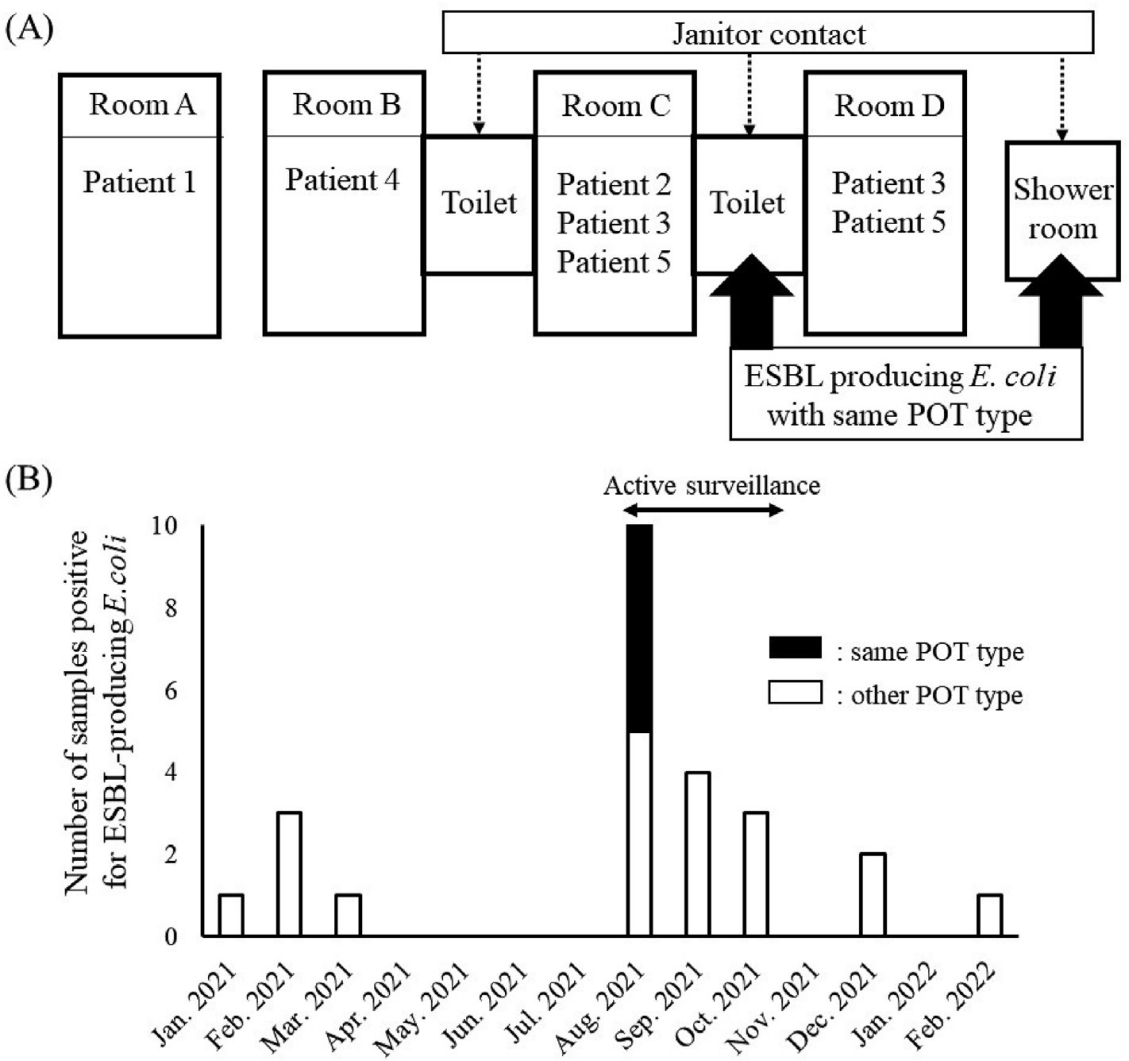
A: Review of the patients' room history. B: Trends of the numbers of samples positive for ESBL-producing *E. coli* detected in the outbreak wards. If the same patient was identified in a different month, it was counted as a duplicate. ESBL; extended spectrum β-lactamase, *E. coli*; *Escherichia coli*, POT; polymerase chain reaction-based open-reading frame typing.

In this case, the patients positive for the outbreak strain had been admitted to a room near the toilet where the outbreak strain was detected. Patient 1 did not use this toilet. However, the same strain was detected in the bath chair in the shower room. Therefore, both toilets and bath chairs were considered potential sources of *E. coli*. A janitor may have been a vector of the outbreak strains detected at these distant locations because of inadequate cleaning procedures. Moreover, the janitor's awareness of contact precautions was limited in our institution. Previous studies reported cases of meticillin-resistant *Staphylococcus aureus* isolated from hospital janitors as opposed to from non-hospital janitors, indicating the possibility of the janitors being vectors of the multidrug resistant strains [7,8]. Our case highlights that improving awareness of contact precautions among janitors is an important factor in preventing the outbreak of multidrug-resistant strains. Janitors are essential to maintaining hygiene in hospitals, but their involvement is invisible until there is an outbreak. Janitors need to be educated on IPC in the same way as other healthcare workers.

Surprisingly, an ESBL-producing *E. coli* strain different from the outbreak strain was detected in eight patients during the active surveillance period. Thus, the number of patients with this strain was a higher than that of the outbreak strain in the ward (Figure 1B). Some studies have reported that the number of carriers of ESBL-producing bacteria in the community is increasing [2], and 3.1% of the healthy people in Japan are carriers of ESBL-producing bacteria [9]. Active surveillance may reveal the true number of carriers. We suggest steps should be taken to prevent the spread of ESBL-producing bacteria in the community.

In conclusion, we reported an outbreak in which the toilet and bath chair were the potential sources of transmission, and that this transmission may have been associated with the inadequate cleaning protocols of the janitors. This report highlights that various environments in a hospital can become potential sources of outbreaks. Therefore, IPC education is crucial not only for the clinical staff but also for non-clinical staff such as janitors.

## Conflict of interest statement

None declared.

## Funding sources

This research did not receive any specific grant from funding agencies in the public, commercial, or not-for-profit sectors.
